# Deciphering community assembly processes of the microbial community in subtropical coastal-estuarine seawater over a 6-year exploration

**DOI:** 10.1093/ismeco/ycaf091

**Published:** 2025-05-30

**Authors:** Yu Wang, Qiongqiong Yang, Qi Chen, Shengwei Hou, Nianzhi Jiao, Qiang Zheng

**Affiliations:** State Key Laboratory of Marine Environmental Science, College of Ocean and Earth Sciences, Institute of Marine Microbes and Ecospheres, Xiamen University, Xiamen, Fujian 361102, PR China; Fujian Key Laboratory of Marine Carbon Sequestration, Xiamen University, Xiamen, Fujian 361102, PR China; College of the Environment and Ecology, Xiamen University, Xiamen, Fujian 361102, PR China; Center for Pan-third Pole Environment, Lanzhou University, Lanzhou, Gansu 730000, PR China; Department of Microbiology, Oregon State University, Corvallis, OR 97331, United States; Department of Ocean Science and Engineering, Southern University of Science and Technology, Shenzhen, Guangdong 518000, PR China; State Key Laboratory of Marine Environmental Science, College of Ocean and Earth Sciences, Institute of Marine Microbes and Ecospheres, Xiamen University, Xiamen, Fujian 361102, PR China; Fujian Key Laboratory of Marine Carbon Sequestration, Xiamen University, Xiamen, Fujian 361102, PR China; State Key Laboratory of Marine Environmental Science, College of Ocean and Earth Sciences, Institute of Marine Microbes and Ecospheres, Xiamen University, Xiamen, Fujian 361102, PR China; Fujian Key Laboratory of Marine Carbon Sequestration, Xiamen University, Xiamen, Fujian 361102, PR China

**Keywords:** community ecology, dispersal, salinity gradient, selection, temporal dynamics

## Abstract

Understanding community assembly in ecosystems is crucial for ecology, yet the interplay between selection and dispersal remains unclear. We examined bacterial and microeukaryotic communities in a dynamic estuarine-coastal ecosystem over six years to investigate the roles of dispersal and environmental selection. Our approach combined species-time relationships (STRs) analysis, a temporal approach focusing on colonization and extinction dynamics, and a process model for community dynamics, revealing insights into the interplay between selection and dispersal along environmental gradients. Both communities showed significant STRs, but their responses varied by taxon and environmental conditions. For bacteria, salinity increased STR exponents, indicating faster richness growth over time, whereas microeukaryotic STR exponents decreased, suggesting distinct assembly mechanisms. Higher salinity reduced bacterial community determinism but heightened it for microeukaryotes, affecting community turnover: microeukaryote turnover decreased with rising salinity due to changes in colonization and extinction, while bacterial turnover increased due to slower dynamics. This study highlights the complex interaction between selection and dispersal, shaped by environmental factors and unique microbial traits, emphasizing the need for tailored conservation strategies in fluctuating coastal ecosystems.

## Introduction

Estuarine-coastal regions, acting as critical transitional zones between land and sea, are integral in linking biogeochemical processes across continents and oceans [1]. These areas are not merely ecotones but function as distinct ecosystems with unique species compositions and transport mechanisms, which govern the dispersal and spatial organization of microbial communities along estuarine-coastal gradients [2]. Salinity gradients, a defining attribute of estuarine-coastal zones, profoundly influence the spatial distribution of physical properties, biota, and biogeochemical cycles [2]. In the context of global change, shifts in precipitation patterns, rising sea levels, and saltwater intrusion may alter coastal salinity, affecting the distribution of marine organisms [2, 3]. Investigating how microbial communities respond to salinity changes is crucial for managing resources in a changing climate [4].

Bacterial and microeukaryotic communities are fundamental components of marine ecosystems, playing crucial roles in structuring food webs and regulating carbon flow through processes such as photosynthesis, predation, and the degradation of dissolved organic matter (DOM) [5, 6]. The marine-freshwater interface has shaped the evolutionary relationships of both macro- and microorganisms over time, with marine and freshwater microbes often displaying distant evolutionary connections [7]. Understanding the ecological processes that drive the community assembly of these microorganisms is a central focus of microbial ecology [8, 9]. Community assembly is governed by both deterministic and stochastic processes. Deterministic processes involve biotic interactions (e.g. competition, predation, mutualism, trade-offs) and abiotic factors (e.g. salinity, temperature, precipitation), collectively influencing community composition through environmental filtering [10, 11]. In contrast, stochastic processes assume species are ecologically equivalent and include random events like birth, death, dispersal, extinction, and speciation [12, 13]. In aquatic environments, both riverine and marine, stochastic processes have been shown to strongly influence the assembly of bacterial and microeukaryotic communities [14–17]. However, deterministic processes can also dominate community assembly at specific spatial scales, especially for bacteria and microeukaryotes [18, 19]. In most cases, prokaryotic and microeukaryotic communities are shaped by a combination of deterministic and stochastic forces [20, 21]. Salinity, in particular, has emerged as a key environmental factor influencing microbial community structure across aquatic and terrestrial ecosystems [22–24]. Despite this, there has been limited research on how shifts in salinity along estuarine-coastal gradients affect the community assembly of bacterial and microeukaryotic populations.

To address these questions, we investigated the temporal dynamics of both bacterial and microeukaryotic communities along salinity gradients in the subtropical estuarine-coastal area of Xiamen Bay, China. Xiamen Island experiences a subtropical climate characterized by a typical humid summer and dry winter, while Xiamen City reflects a fast-developing economic region with enhanced anthropogenic activity. Previous studies in Xiamen Bay have revealed clear spatiotemporal variations in environmental conditions and microbial communities [25, 26]. Our prior work has demonstrated the important roles of microbial communities in mediating the ecosystem function in this estuarine-coastal area [27]. Nevertheless, to understand seasonal changes in biodiversity and ecosystem functioning, it is necessary to elucidate temporal alterations in the structure of bacterial and microeukaryotic communities, their correlation with environmental parameters, and the relationship between microbial populations based on their co-occurrence patterns and functional traits [28]. Despite this importance, it remains unclear how salinity specifically influences the assembly mechanisms of bacterial and microeukaryotic communities. A recent study shows that salinity significantly affects the assembly mechanisms of microbial communities in lakes, exhibiting different responses from bacterial and microeukaryotic communities [29]. Xiamen Bay represents an ideal system to investigate the relative contributions of dispersal and environmental filtering in microbial community assembly processes. Frequent freshwater inputs, anthropogenic activities, and periodic disturbances (e.g. floods and typhoons) generate highly dynamic environmental conditions that strongly affect microbial dispersal patterns and environmental filtering. Understanding how these processes shape microbial communities in such dynamic coastal waters is crucial for predicting community responses to ongoing environmental changes. According to the size-plasticity and size-dispersal hypotheses [30], which propose that ecological determinism intensifies with organism size, we hypothesized that bacterial and microeukaryotic communities would exhibit distinct assembly mechanisms in response to temporal variation and salinity gradients, reflecting their differing ecological traits and environmental sensitivities. Our study aims to explore the mechanisms underlying the observed dynamics and to discern the varying effect of these processes on environmentally driven temporal variability in microbial communities.

## Materials and methods

### Sampling information

Three sampling sites (S03, S05, and S07) were monthly (except February) investigated in the coastal area surrounding Xiamen Island (~24°N, ~118°E) from the year 2015 to 2021 (Fig. S1). Sampling was paused in February due to logistical constraints during the Chinese Lunar New Year holidays. Water samples were collected at approximately 1 m in depth at all the stations. Water temperature, salinity, and pH were measured at the time of sample collection using a YSI Pro Plus Multi-parameter (Professional Plus, USA). All the seawater samples were pre-filtered through a 20-μm pore size filter to remove large eukaryotic organisms and large particles. Samples (1 L) were filtered through a 0.22-μm polycarbonate filter (Millipore, Billerica, MA, USA) and stored at −80°C for DNA extraction. Inorganic nutrient concentrations including phosphate (PO_4_) and nitrate+nitrite (NO_x_) were measured using the PowerMon Kolorimeter AA3 (Bran+Luebbe, Charlotte, NC, USA) following spectrophotometric methods described in a previous study [31].

### DNA extraction and bioinformatics

The total seawater DNA was extracted from the filters using the phenol-chloroform-isoamyl alcohol method as detailed by Wang et al., (Wang et al., 2017). 16S rRNA gene primer (V4-V5) is 515F (5’-GTGCCAGCMGCCGCGGTAA-3′) and the reverse primer 907R (5’-CCGTCAATTCMTTTRAGTTT-3′) [27]; while 18S rRNA gene primer (V4) is 5’-CCAGCAGCCGCGGTAATTCC-3′ and 5’-ACTTTCGTTCTTGATTAA-3′ [32]. Sequencing libraries were generated using an NEB Next Ultra DNA library prep kit (New England Biolabs, Waltham, MA, USA) for Illumina following the manufacturer’s recommendations. The library was sequenced on an Illumina MiSeq platform (Suzhou Genewiz Biotechnology Co., Ltd., Suzhou, China), generating paired end reads (2 × 250 bp). Amplicon sequence variants (ASVs) were clustered based on high-quality sequences (Q value of > Q20 and length of >50 bp) with a 99% similarity cutoff using deblur (v2). Reads of average 280 bp (16S rRNA) and 229 bp (18S rRNA) were obtained, respectively. The ASVs were taxonomically classified based on the SILVA database (version 138.2). All chloroplasts, mitochondria, and metazoan sequences were discarded for the following analysis. Data were normalized by rarefying the reads of all samples to the minimum number of reads across the data set (bacteria, 15 982 reads; microeukaryotes, 12 211 reads). The resulting ASV tables contained 5336 bacteria and 5671 microeukaryotes. For community-related analysis, rarefaction at the same depth was repeated 100 times [29].

### Species time relationship

In ecology, the relationship between species richness and time (species time relationship, STR) is often described by the power-law equation *S* = *c*T*^w^*, or its logarithmic equation, which is analogous to the power law of species-area relationship [33]. *S* is the number of observed species within the length of time T, *c* is an empirically derived constant, and *w* is the STR exponent, i.e. a measure of the temporal scaling rate of species richness [34, 35]. The geometric mean of richness was calculated. The significance test of the model is based on 1000 times permutations.

### Colonization and extinction rates

First, we implemented the simplest stochastic model that underpins the theory of island biogeography [36–38]. This dynamic model elucidates the average level of richness and its variation in the study site (or “island”) in terms of colonization and extinction processes, alongside the total number of potentially colonizing species in the regional pool, also referred to as metacommunity richness [39]. This model can be derived from an ensemble of single-species models of presence-absence dynamics, under the assumptions of both species independence and equivalence [36, 40]. Therefore, we were able to infer the model parameters for the dynamics of the whole community from presence-absence temporal data. This approach allows us to separately infer the colonization-extinction dynamics for bacterial and microeukaryotic communities, characterizing each community with its own colonization-extinction pair. To derive colonization (*c*) and extinction (*e*) rates based on the observed data, given the irregular sampling intervals of bacterial and microeukaryotic communities, we used *irregular_single_dataset* function from *island* package [39, 41]. Meanwhile, a characteristic time for each station was calculated [42]. This characteristic time is based on *c* and *e* (1/(*c* + *e*)), which is expressed in days and is inversely related to the turnover rate of the community [42]. Therefore, lower characteristic times indicate more dynamic (high turnover) communities, while higher characteristic times indicate greater stability (lower turnover). Additionally, the effect of salinity (standardized contribution of salinity) on colonization and extinction was evaluated using the equations described in a previous study [29]. Briefly, the contributions of salinity to bacterial colonization (*c*) and extinction (*e*) rates were evaluated separately for each sampling site by explicitly incorporating salinity as an environmental factor into the dynamic model. This allowed us to independently estimate both the direction (positive or negative) and magnitude of salinity effects at each station. Subsequently, standardized model parameters were calculated to quantify the responses of colonization and extinction rates to salinity variations. Negative contributions indicated that increased salinity reduced colonization or extinction rates, resulting in lower community turnover (i.e. longer characteristic times), which was consistently observed across all sampling stations.

### Determinism based on a novel process model

Second, we used a recently developed process model-based approach to quantitively delineate community assembly mechanisms [43]. This method combined the consumer-resource model with a neutral model in stochastic differential equations. Salinity was used within the consumer-resource model. Briefly, the determinism of each taxon was estimated at each time point under the combined consumer-resource model with the neutral model. Then, the community-level determinism was calculated as the mean determinism among taxa, either weighted by the relative abundance of each taxon (weighted determinism) or not (unweighted determinism). The process model integrates consumer-resource dynamics with neutral theory through two key assumptions [43]. First, environmental filtering operates via salinity-mediated resource competition following Monod kinetics, where species' growth rates depend on shared limiting resources. Second, stochastic neutral processes emerge through demographic noise and dispersal events modeled as Wiener processes in the stochastic differential equations. While interspecific interactions (e.g. competition coefficients) are implicitly captured through overlapping resource utilization profiles, explicit predator–prey dynamics or mutualistic interactions are not parameterized in this framework. This parsimonious structure allows separation of environmental selection (deterministic terms) from stochastic dispersal-drift effects (random walk terms).

### Statistical analyses

The significant difference in coefficient of variation (CV) of salinity and temperature was tested using a modified signed-likelihood ratio test (SLRT) with 1000 times simulation runs in *cvequality* package in R [44]. The influence of environmental variables on the microbial composition and structure was preliminarily assessed by correlation approaches, Mantel tests (Pearson's correlation), and nonmetric multidimensional scaling (NMDS) ordinations based on Bray-Curtis dissimilarities calculated for each pair of samples after Hellinger standardization using *base* and *vegan* packages in R [45, 46]. Three nonparametric dissimilarity tests, including multi-response permutation procedure (MRPP), analysis of similarities (ANOSIM), and nonparametric multivariate analysis of variance (adonis) in the *vegan* package in R [45], were used to compare differences of microbial community composition based on Bray-Curtis dissimilarities after Hellinger standardization. Kruskal–Wallis test and Dunn Kruskal–Wallis test were conducted using *base* and *FSA* packages [46, 47]. Spearman or Pearson correlation and linear regression were calculated using the *base* package in R [46].

## Results

### Beta-diversity patterns across environmental scales

**Figure 1:**
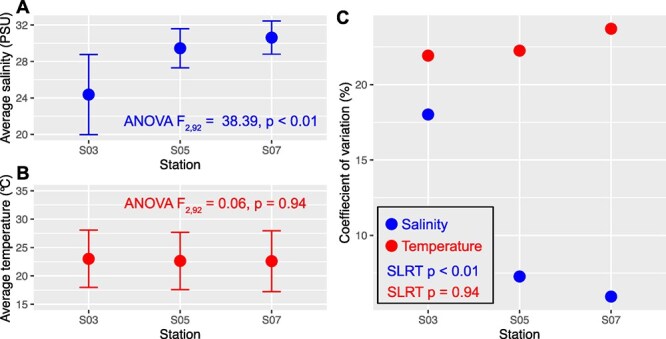
Average salinity (A) and temperature (B) and their coefficient of variations (CV) in S03, S05, and S07 (C). Differences in average salinity and salinity among the three stations were tested using analysis of variance (ANOVA). The equality of CV among three stations was tested by a modified signed-likelihood ratio test (SLRT) with 1000 times simulation runs.

**Figure 2:**
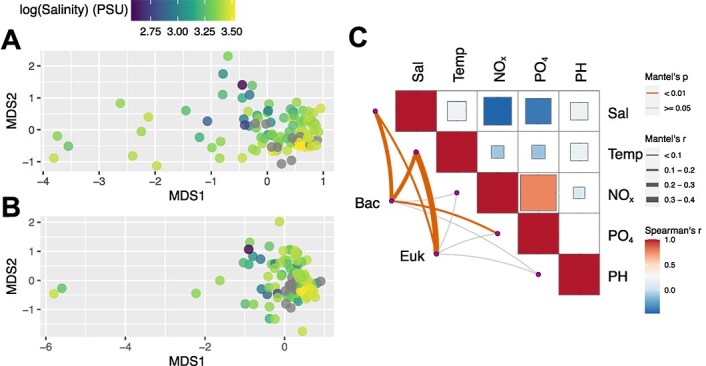
Community structure variation along the salinity and relationships between the environmental variables and community structure. (A and B) The non-metric multidimensional scaling (NMDS) representation of the Bray–Curtis dissimilarity among samples for bacteria (A) and microeukaryotes (B). The dot color indicates log-transformed salinity. (C) Pairwise comparisons of environmental variables are shown at the upper right, with a color gradient representing Spearman’s correlation coefficient. Only significant correlation (*P* < .05) was shown. Bacteria (Bac) and microeukaryote (Euk) compositions were correlated to each environmental variable by Mantel tests. The line width represents Mantel’s r statistic for the corresponding correlation, and the line color means that significances are tested based on 999 permutations. Temp, temperature; Sal, salinity; NO_x_, NO_3_ + NO_2_ concentration; PO_4_, phosphate concentration.

In the estuarine-coastal seawaters, there was an observed increase in average salinity from S03 to S07 (Fig. 1A), while the temperatures were similar (Fig. 1B). The coefficient of variation (CV) of salinity in S03 exceeded that of S05 and S07 (Fig. 1C, SLRT *P* < .001), suggesting a pronounced influence of the Jiulong River on salinity dynamics in S03. In contrast, there was no significant difference in CVs between S05 and S07 (Fig. 1C, SLRT *P* = .94). Statistical analyses revealed significant differences in the composition of both bacterial and microeukaryotic communities among the three stations (Table 1). Consequently, salinity was selected as a proxy of environmental variability due to its pivotal role in shaping microbial community composition and its covariation with other tested environmental variables (Fig. 2C). Furthermore, S03 showed a distinct community structure from S05 and S07, whereas the community compositions were similar between S05 and S07 (Table 1). NMDS results showed considerable variability in microbial community composition along the salinity changes (Fig. 2A, B). Dissimilarity between pairs of samples revealed stronger correlations with pairwise salinity and temperature differences for both bacteria and microeukaryotes (Fig. 2C). Notably, Sørensen dissimilarities of bacterial communities increased from S03 to S07, whereas those within microeukaryotes decreased from S03 to S07 (Fig. S2). Furthermore, the high correlation between the beta-diversity patterns of the two microbial domains (Mantel’s r = 0.85 and *P* < .001) supported their equivalent responses to the tested environmental variables.

**Table 1:** Significance tests of the networked communities among stations S03, S05, and S07.

	MRPP		ANOSIM		Adonis	
	Δ	*p*	R	*p*	F	*p*
Bacteria						
S03-S05-S07	0.652	.029	0.123	.001	3.898	.001
S03-S05	0.651	.001	0.120	.001	3.797	.001
S03-S07	0.650	.001	0.233	.001	6.601	.001
S05-S07	0.649	.159	0.020	.076	1.326	.153
Microeukaryotes						
S03-S05-S07	0.769	.016	0.042	.004	1.638	.009
S03-S05	0.770	.030	0.049	.018	1.721	.018
S03-S07	0.772	.001	0.076	.001	2.265	.002
S05-S07	0.764	.654	0.002	.319	0.921	.527

Significant effects are indicated in bold.

Abbreviation: MRPP, multi-response permutation procedure; ANOSIM, analysis of similarity; Adonis, permutational multivariate analysis of variance.

### STRs and dynamic behavior

**Figure 3:**
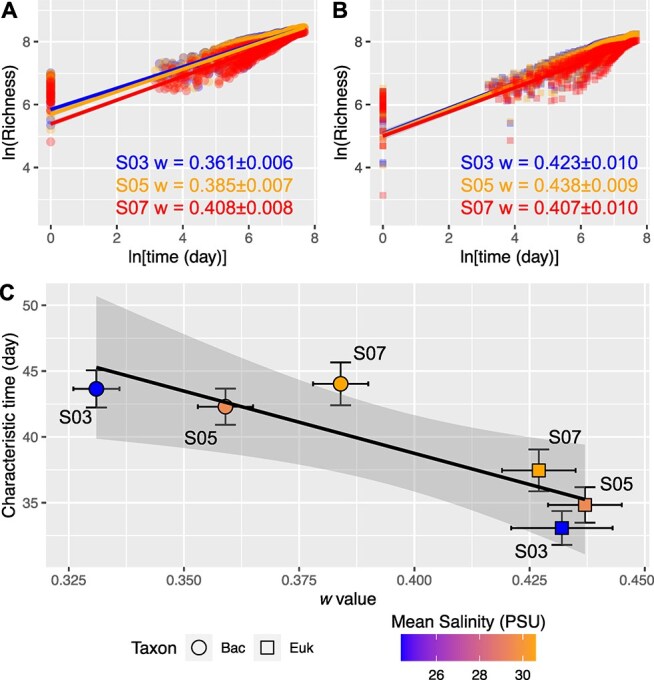
Species–time relationships (STRs) of bacteria (A) and microeukaryote (B) and the correlation of characteristic time of bacterial and microeukaryotic communities with STR’s *w* value (C). (A and B) The fit method is a non-linear regression model, with a power model: *S* = cT*^w^*. The geometric mean of richness was calculated. For bacteria, S03, w = 0.347 ± 0.006, adjusted R^2^ = 0.90, *P* < .001; S05, w = 0.373 ± 0.006, adjusted R^2^ = 0.90, *P* < .001; S07, w = 0.396 ± 0.008, adjusted R^2^ = 0.89, *P* < .001. For microeukaryote, S03, w = 0.423 ± 0.010, adjusted R^2^ = 0.83, *P* < .001; S05, w = 0.438 ± 0.009, adjusted R^2^ = 0.87, *P* < .001; S07, w = 0.407 ± 0.010, adjusted R^2^ = 0.82, *P* < .001. (C) Pearson’s correlation between STR’s w value and characteristic time was applied (r = −0.88, *P* = .02). The error bar indicates the standard deviation for the characteristic day and the standard error for the w value. Mean Sal indicates the mean salinity in each station.

Our results revealed that the data from bacteria and microeukaryotes fitted the logarithmic equation very well in each station (R^2^ = 0.82–0.90, *P* < .001; Fig. 3A, B), suggesting that there are strong STRs in both bacteria and microeukaryote in each station. Notably, the microeukaryotic community exhibited higher *w* values compared to the bacterial community. Specifically, *w* values for the bacterial community increased along the salinity gradient (S03, 0.347 ± 0.006; S05, 0.373 ± 0.006; S07, 0.396 ± 0.008; ANOVA, *P* < .01), while a decrease in *w* value was observed for the microeukaryotic community across the three stations (S03, 0.423 ± 0.010; S05, 0.438 ± 0.009; S07, 0.407 ± 0.010; ANOVA, *P* < .01). Our results highlighted salinity as a significant driver, promoting temporal scaling rates of marine bacterial richness but decreasing microeukaryotic richness.

**Figure 4:**
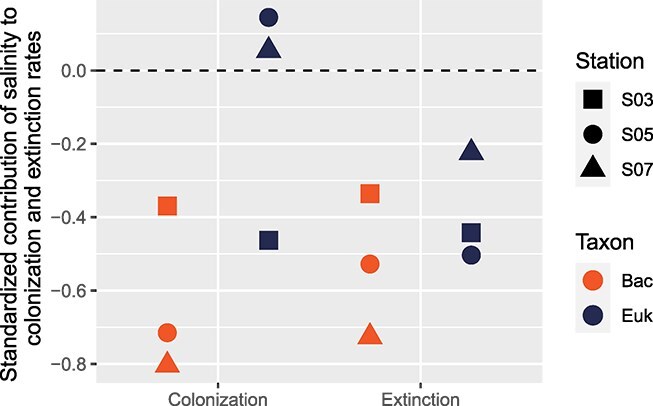
Relative effect of salinity on colonization and extinction rates. The standardized contribution (compared to basal colonization/extinction) is shown for each of the microbial groups.

Characteristic times, indicators of community stability over time, were higher for bacterial communities compared to microeukaryotic communities (Fig. 3C), suggesting that bacterial communities were more stable than microeukaryotic communities. Moreover, an increase in characteristic times with salinity was observed for microeukaryotes, but only a limited change for bacterial communities (Fig. 3C). Additionally, a significant correlation between *w* values and characteristic time was evident (Pearson’s correlation, r = −0.89 and *P* = .04), which suggests that characteristic time could represent the turnover rate changes between the bacterial and microeukaryotic communities. Furthermore, we assessed the impact of salinity variations on expected richness within each station and each taxon by comparing the product of the standardized model parameters capturing the effect of salinity on colonization and extinction rates. Salinity variations exhibited differential effects on bacterial and microeukaryotic community dynamics (Fig. 4). For bacteria, a uniform effect of salinity on colonization and extinction rates were found along the salinity gradient. The contribution of salinity to bacterial colonization and extinction rates was negative for all stations and increased with the salinity, which indicated a decrease in colonization and extinction rates as salinity increased. Conversely, for microeukaryotes, the effect of salinity differed between colonization and extinction, with positive values observed for colonization at high salinity and negative values at low salinity. Salinity contributed negatively to extinction rates for microeukaryotes, with this contribution decreasing as salinity increased. The observed lower determinism and shorter characteristic times at the river mouth station, which experienced higher salinity variation (CV), suggest that dispersal-driven stochastic processes play a substantial role at this highly disturbed location. Frequent disturbances (e.g. floods and typhoons) likely enhance microbial dispersal, increasing stochasticity in community assembly. Conversely, sites with relatively stable salinity conditions exhibited higher determinism, indicating stronger environmental filtering and a reduced influence of dispersal-driven stochasticity. These findings highlight the dynamic balance between dispersal processes and environmental selection in shaping microbial communities across different sites in Xiamen Bay. While colonization and extinction rates reflect the dynamic nature of seawater environments, it is the balance between these processes that ultimately governs the average community richness at equilibrium. This balance shifted along the salinity gradient, with bacterial and microeukaryotic communities responding differently. As salinity increased, the differential contributions of colonization and extinction led to distinct variations in metacommunity richness between these two groups.

### Species and community determinism

**Figure 5:**
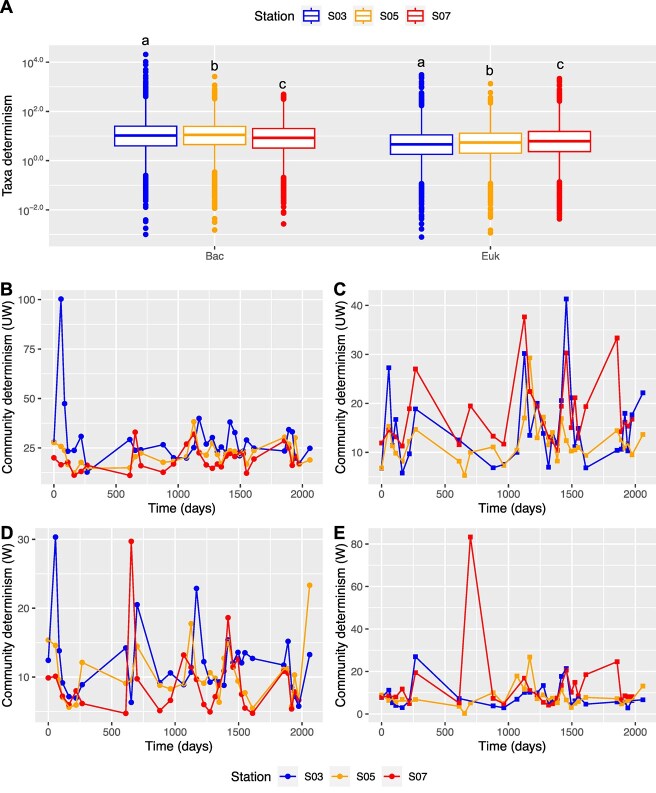
Species-level and community-level determinism. (A) Predicted determinism across taxa from bacteria and microeukaryotes. Different letters above boxed indicate the significant difference by Dunn Kruskal–Wallis test with an adjusted *P* < .05. (B-E) Comparisons of the predicted unweighted and weighted community-level determinism of bacterial communities (B and D) and microeukaryotic communities (C and E) among the stations.

The analysis of taxa determinism at specific time points showed significant differences in mean determinism between bacterial and microeukaryotic communities (mean determinism: 24 vs 15; *P* < .0001 by the Kruskal–Wallis test) (Fig. 5A). This result suggested a stronger selection pressure on bacterial communities compared to microeukaryotic communities. Moreover, the mean taxa determinism of bacterial communities exhibited a decreasing trend from S03 to S07 (mean determinism: S03, 39; S05, 22; S07, 19; *P* < .0001 by the Kruskal–Wallis test), with significant differences observed among the three stations by the Dunn Kruskal–Wallis test (S03 vs S05, adjusted *P* = .04; S03 vs S07, adjusted *P* < .0001; S05 vs S07, adjusted *P* < .0001). In contrast, the mean taxa determinism of microeukaryotic communities decreased from S03 to S05 but significantly increased to S07 (mean determinism: S03, 15; S05, 12; S07, 17; *P* < .0001 by the Kruskal–Wallis test), with all adjusted *P*s < .0001 by the Dunn Kruskal–Wallis test.

The community-level determinism was further derived by aggregating the determinism of co-occurring taxa within the community (Fig. 5B–E). Both abundance-weighted determinism and unweighted community determinism for bacterial and microeukaryotic communities were significantly different among the three stations (for bacteria, *P*s = .01319 and < .0001; for microeukaryotes, *P*s = .0005 and .0169 by Kruskal–Wallis test). Subsequent Dunn Kruskal–Wallis test identified significant differences in abundance-weighted determinism and unweighted community determinism for bacterial communities between S05 and S07 (adjusted *P*s = .03 and .04), as well as S03 and S07 (adjusted *P*s = .03 and .0003). However, no significant differences were observed between S03 and S05 (adjusted *P*s = .84 and .11). For microeukaryotes, only abundance-weighted determinism between S03 and S07 exhibited a significant difference (adjusted *P* = .0098), with no significant difference between S03 and S05 (adjusted *P* = .17) or S05 and S07 (adjusted *P* = .22). In contrast, significant differences in abundance-unweighted determinism were found between S03 and S05, as well as S05 and S07 (S03 vs S05, *P* = .005; S03 vs S05, adjusted *P* < .0001), with no significant difference between S05 and S07 (adjusted *P* = .07). Additionally, significantly positive correlations were observed between temperature and both weighted and unweighted community determinism of bacterial and microeukaryotic communities (Fig. S3). In summary, these results indicate a more pronounced seasonal selective pressure on microeukaryotes but a decreased one on bacteria in environments characterized by elevated salinity.

## Discussion

The assembly of microbial communities, particularly in coastal environments, presents a complex interplay between deterministic and stochastic processes. Understanding how selection, dispersal, and drift shape community composition is central to microbial ecology [48], yet many of these mechanisms remain poorly understood due to the inherent complexity of microbial systems. Additionally, the distinctive features of microbial systems have historically constrained temporal community studies to descriptive analyses of diversity and compositional changes, leaving the underlying mechanisms driving community assembly shifts largely unexamined [29]. In this study, we have employed a temporal analysis integrating extinction-colonization dynamics and a novel process model-based method, providing valuable insights into the mechanistic pathways through which selection and dispersal converge to shape the assembly of communities.

Understanding temporal scaling and its underlying mechanisms in the context of climate change is a critical challenge in ecology and global change biology. However, studies exploring the relationship between species diversity and time in microbial communities remain scarce, particularly in marine environments [49–51]. The significant STR observed in both bacterial and microeukaryotic communities suggests their turnover variations in the coastal area (Fig. 3A, B). Notably, microeukaryotic communities typically exhibited higher turnover rates (*w*, 0.407–0.438) compared to bacterial communities (0.373–0.396), suggesting more rapid seasonal divergence in microeukaryotic communities. This finding is further reinforced by the significantly negative correlation between turnover rates and characteristic time, which indicates community stability (Fig. 3C). Furthermore, turnover rates vary across different organisms [51]. The *w* values of bacteria and microeukaryotes (0.373–0.396 and 0.407–0.438) in coastal seawaters are substantially higher than those observed in plants and animals (0.21–0.38) [51]. The *w* value for bacteria aligns closely with that of soil bacteria (0.39), while the *w* value for microeukaryotes is lower than that of soil fungi (0.64) [51]. Notably, caution should be taken when directly comparing microbial community dynamics observed in our study to those of plants or animals, as differences in sampling intervals, study duration, and organismal characteristics (such as generation time and dispersal capability) may significantly influence observed community turnover rates and assembly processes. Nevertheless, these results are consistent with the predictions of metabolic theory, which suggests that organisms with higher metabolic rates experience faster ecological and evolutionary processes [52]. The observed differences in salinity responses between bacterial and microeukaryotic communities may be partly attributed to inherent physiological and ecological traits, such as metabolic rate and cell size [53]. Bacteria typically possess higher metabolic rates and smaller cell sizes [51], enabling rapid physiological responses to changing osmotic pressures driven by salinity fluctuations. In contrast, microeukaryotes often have larger cell sizes and comparatively slower metabolic processes, potentially limiting their ability to rapidly adjust to salinity shifts [23], thereby contributing to the observed differences in community turnover rates and stability.

The influence of environmental changes on the temporal scaling rate of microbial communities has been documented across various environments [51]. Although temperature and nutrient concentrations are important factors influencing microbial communities (Fig. 2C), our long-term monitoring indicates that temperature remained relatively stable over the sampling, and salinity exhibited strong covariance with nutrient levels (Figs. 1 and 2C). Therefore, salinity emerged as the primary environmental driver in our system, enabling us to effectively capture nutrient-related effects through its analysis. We found that the salinity significantly altered the taxonomic composition of both bacterial and microeukaryotic communities (Fig. 2C). This underscores the susceptibility of bacterial and microeukaryotic communities to salinity variations, revealing their limited resilience when confronted with such environmental shifts. Specifically, the temporal scaling rates of bacterial communities increased with rising salinity, suggesting accelerated divergence at high salinity stations. Conversely, turnover rates of microeukaryotic communities generally declined along the salinity gradient, illustrating distinct responses between these two microbial domains to changes in salinity. The differences in turnover rates between bacteria and microeukaryotes driven by variations in metabolism and cell size [53], suggest that distinct mechanisms govern their responses to environmental changes, illustrating the complexity of community assembly.

This complexity is further compounded by the ecological differentiation among microbial domains, which have varying sensitivities to environmental fluctuations and differing dispersal capabilities [30], making the unraveling of ecological drivers in microbial ecology a significant challenge [54]. In our study, along the salinity-induced stress gradient, we consistently observed shifts in the balance of community assembly processes between bacterial and microeukaryotic domains, highlighting the distinct ways in which they respond to environmental pressures. The dynamic environmental conditions in coastal estuaries, such as floods, wastewater inputs, and typhoons, further complicate the processes of community assembly, as they can have profound impacts on microbial communities. For example, occasional typhoon events have been shown to enhance stochastic effects in microbial community assembly [16, 55]. This highlights how temporal variations in environmental factors, such as extreme weather events, can significantly influence the assembly processes in highly dynamic coastal waters. This is consistent with the lower determinism and shorter characteristic time we observed in microeukaryotic communities at the river mouth station, where high salinity variation (CV) was present (Figs. 1 and 3). Moreover, the characteristic time for microeukaryotic communities showed a gradual increase from low to high salinity levels (Fig. 3C), indicating that environmental filtering on microeukaryotes increased along with the increased salinity. Strong environmental filtering often results in lower community turnover rates, as selective pressures consistently favor specific taxa adapted to prevailing environmental conditions [56–58], thus stabilizing community composition.

Such domain-specific responses to environmental filtering were clearly reflected in the contrasting assembly patterns we observed between bacterial and microeukaryotic communities along the salinity gradient. In an urban reservoir, influence of selection on microeukaryotes increased as salinity increased [23]. In contrast, bacterial communities displayed an opposite pattern, with a slight increase in characteristic time with increasing salinity but a marked decrease in determinism (Fig. 5). This difference can likely be attributed to decreased nutrient concentrations, including NO_x_ and PO_4_, which are closely linked to bacterial community composition, as observed in both our study (Fig. 2C) and previous studies [26, 27]. In addition, the bioavailability of DOM decreased with salinity, exhibiting a positive correlation with nutrient concentrations, indicating decreased selection pressure on bacterial communities [27]. During the summer months, DOM molecular composition at station S03 differed significantly from other stations [27], pointing to increased selection pressure on bacterial communities during this period. Consistent with these findings, significant positive correlations between community determinism and temperature further support the notion that selection processes in microbial community assembly are heightened during summer (Figs. 5B and S3). However, different correlations of determinism with temperature across stations implied salinity variation might affect the responses of microbial communities to seasonal environmental change. For microeukaryotes, as salinity-induced selective pressures intensified, the influence of drift diminished, leading to reduced beta diversity among samples (Fig. S2). This decline in beta diversity is likely due to the need for physiological adaptations, which are limited to specific groups capable of surviving in high-salinity environments [56–58]. Furthermore, the metabolic demands required to sustain energy-intensive adaptations create salinity thresholds for certain processes, ultimately reducing species richness as salinity increases [56, 57, 59]. This trend was more pronounced in microeukaryotes than in bacteria, likely reflecting differences in their metabolic flexibility and physiological responses to salinity variations [60, 61]. The varying responses of coexisting microbial groups to the same assembly factors have been observed across numerous environments [18, 30, 62–66], highlighting the complexity of microbial community dynamics in response to environmental gradients.

The dynamics of microbial communities can also reflect their stability. The observed higher characteristic time indicates greater stability in bacterial communities compared to microeukaryotic communities. This stability is reflected in a slower temporal scaling rate of change in bacterial community composition, suggesting a stronger resistance to environmental fluctuations in saline conditions. This trend, previously observed in artificial environments, is associated with heightened selective pressures [49]. This is further supported by the increased determinism for bacteria than microeukaryotes (Fig. 5) and significant correlations of mean species determinism with colonization and extinction rates in our study (Spearman correlation, *P*s = .04 and .02). The increase in temporal stability, along with the homogenization of community composition (Table 1 and Fig. S2), suggests a gradual shift from stochastic to niche-based community assembly. This pattern mirrors observations from primary succession under intensifying selective pressures [67] and aligns with our findings from the process model-based approach (Fig. 5). However, the responses of bacterial and microeukaryotic communities to the salinity gradient differed significantly. In bacteria, the similar effects of salinity on both colonization and extinction rates led to overall stability in species richness along the gradient (Fig. 4). In contrast, for microeukaryotes, extinction rates decreased more than colonization rates, resulting in increased species richness at higher salinities. This divergence likely arises because salinity exerts a stronger control over microeukaryote colonization success compared to extinction processes [29]. Bacteria, however, appeared less constrained by this limitation, likely due to their greater adaptability and the more pronounced shifts in species composition. This may be attributed to bacterial genetic diversity, horizontal gene transfer, and metabolic flexibility that enhance their ability to thrive under varying salinities [68, 69]. A more detailed investigation of this phenomenon, through more frequent sampling and across larger environmental gradients, could uncover transient species that appear briefly. Such an approach could offer valuable insights into potential colonizing species that have narrow windows for successful establishment.

Several limitations of our modeling approach warrant consideration. First, while the consumer-resource component captures environmental filtering through salinity-mediated resource competition, it simplifies complex biotic interactions into implicit competition coefficients [43]. Predation, viral lysis, and symbiotic relationships, known to influence microbial community assembly [70], are not explicitly modeled but may be partially represented in the stochastic terms. Second, the neutral component accounts for dispersal limitation through the metacommunity connectivity parameter (*m*) but assumes homogeneous dispersal probabilities across taxa. Recent studies suggest microbial dispersal kernels may vary with cell size and motility [30], which could be incorporated through taxon-specific m values in future implementations. Nevertheless, our model's strength lies in its ability to disentangle the dominant assembly mechanisms operating at the community level [43], providing a quantitative framework for comparing microbial domains under environmental change.

## Conclusions

Overall, our findings highlight that the metacommunity structure in coastal seawaters arises from a complex interplay between selection processes and dispersal mechanisms, with varying degrees of influence across the salinity gradient. The distinct ecologies of bacterial and microeukaryotic communities led to differing impacts on community turnover rates and biodiversity, shaped by the interaction between selection and dispersal. Selection processes operated differently between the two microbial groups, with environmental filtering playing a greater role in constraining the extinction of microeukaryotes, underscoring the environmentally driven temporal variability in microbial assembly. Moreover, the differential colonization-extinction trade-offs among microbial taxa contribute to their distinct ecological roles within the food web [39]. A deeper understanding of these trade-offs can enhance our knowledge of how diverse microbial communities maintain their functional roles within ecosystems. The accelerating impacts of climate change pose significant threats to coastal ecosystems, ranging from biodiversity loss to the disruption of coastal agroecosystem management [71, 72]. For instance, reduced determinism in bacterial communities under high salinity could impair organic matter degradation because decreased deterministic selection enhances stochastic assembly processes, increasing variability and reducing stability within microbial functional groups responsible for organic matter degradation. This instability, coupled with inherently lower bioavailability of DOM in higher salinity environments [27], collectively diminishes the microbial community’s capacity for efficient DOM degradation. Similarly, microeukaryotic homogenization can disrupt trophic interactions by reducing functional diversity and the associated resilience of trophic networks. Proactive management strategies, such as preserving freshwater inflows to buffer salinity extremes, could enhance microbial resilience, maintaining essential ecosystem services and stability under anticipated climate-driven salinity fluctuations. Therefore, future research focused on marine microbial assembly mechanisms over broader spatiotemporal scales is crucial for informing effective conservation efforts and developing targeted ecosystem management strategies [73].

## Supplementary Material

20220318_Supplementary_information_v1_3_ycaf091

## Data Availability

The 16S and 18S rRNA gene amplicon sequences data were deposited to the National Center for Biotechnology Information (NCBI) Sequence Read Archive through BioProject PRJNA1108889. The environmental attributes dataset is deposited in figshare with the doi: 10.6084/m9.figshare.25772259.
