# Supplementary material for: Deciphering community assembly processes of the microbial community in subtropical coastal-estuarine seawater over a 6-year exploration
Source: ISME Commun. 2025 May 30;5(1):ycaf091. doi: 10.1093/ismeco/ycaf091 (PMC12203064; doi:10.1093/ismeco/ycaf091)
Supplement: 20220318_Supplementary_information_v1_3_ycaf091 [file 20220318_supplementary_information_v1_3_ycaf091.pdf]

Supplementary information for Deciphering community assembly processes of the microbial community in subtropical coastal-estuarine seawater over a six-year exploration

Yu Wang[1,2,3], Qiongqiong Yang[4], Qi Chen[5], Shengwei Hou[6], Nianzhi Jiao[1,2], Qiang Zheng[1,2]

[1]State Key Laboratory of Marine Environmental Science, College of Ocean and Earth Sciences, Institute of Marine Microbes and Ecospheres, Xiamen University, Xiamen, 361102, PR China.

[2]Fujian Key Laboratory of Marine Carbon Sequestration, Xiamen University, Xiamen, 361102, PR China.

[3]College of the Environment and Ecology, Xiamen University, Xiamen, 361102, PR China.

[4]Center for Pan-third Pole Environment, Lanzhou University, Lanzhou 730000, PR China.

[5]Department of Microbiology, Oregon State University, Corvallis, OR 97331, USA.

[6]Department of Ocean Science and Engineering, Southern University of Science and Technology, Shenzhen, 518000, PR China.

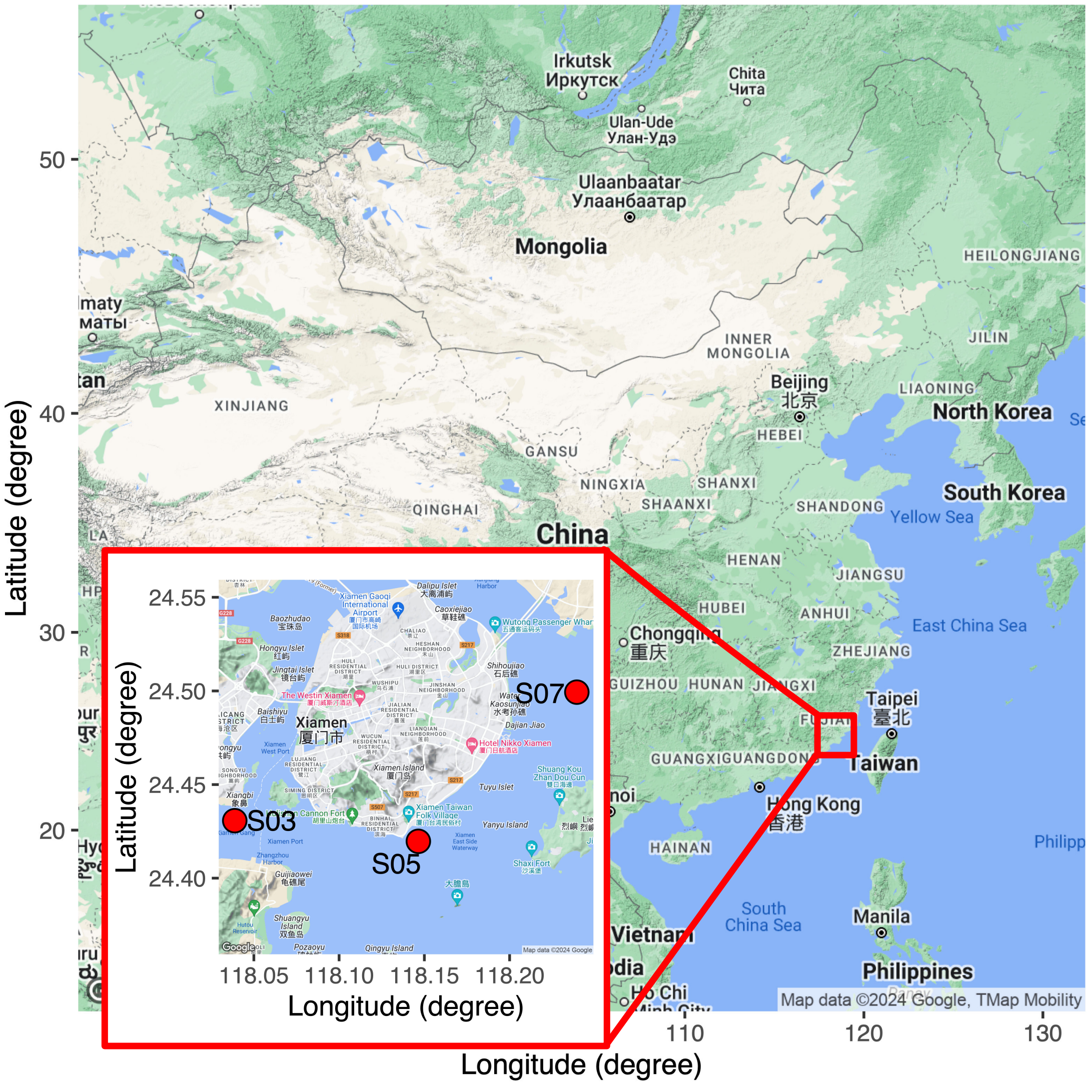

Figure S1 Sampling stations in the coastal area around Xiamen City, China.

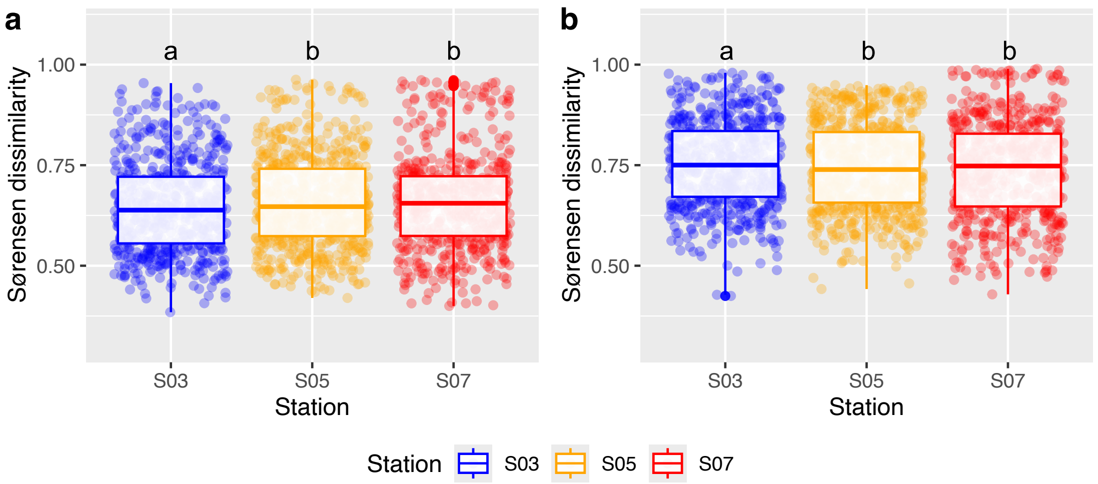

Figure S2 The Søresen dissimilarities between samples belonging to the same station for bacterial (a) and microeukaryotic (b) communities. The different letters indicate the significant differences which were tested by Kruskal-Wallis with p-value < 0.10.

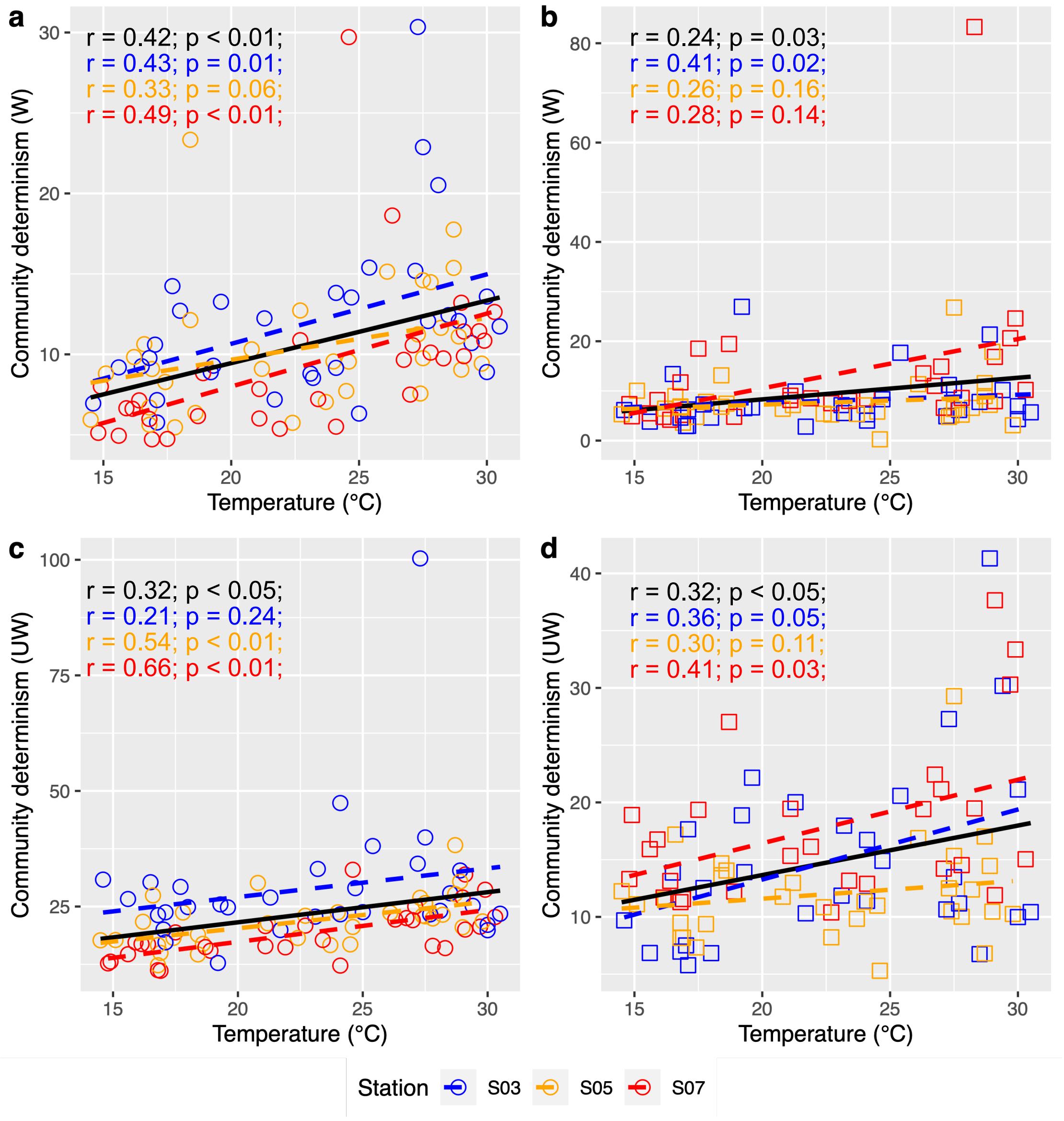

Figure S3 Linear regression of community-level determinism in bacterial communities (a and c) and microeukaryotic communities (b and d) against temperature. The predicted weighted (a and b) and unweighted (c and d) were shown. The black line indicates the general regression in all stations, while the blue, orange, and red dashed lines indicate the linear regression in each station.
